# Machine-learning based reconstructions of primary and secondary climate variables from North American and European fossil pollen data

**DOI:** 10.1038/s41598-019-52293-4

**Published:** 2019-11-01

**Authors:** J. Sakari Salonen, Mikko Korpela, John W. Williams, Miska Luoto

**Affiliations:** 1 Department of Geosciences and Geography, University of Helsinki, PO Box 64, FI-00014 Helsinki, Finland; 2 Environnements et Paléoenvironnements, Océaniques et Continentaux, UMR 5805, Université de Bordeaux, Pessac, France; 3 Department of Geography and Center for Climatic Research, University of Wisconsin–Madison, Madison, WI 53706 USA

**Keywords:** Palaeoclimate, Palaeontology

## Abstract

We test several quantitative algorithms as palaeoclimate reconstruction tools for North American and European fossil pollen data, using both classical methods and newer machine-learning approaches based on regression tree ensembles and artificial neural networks. We focus on the reconstruction of secondary climate variables (here, January temperature and annual water balance), as their comparatively small ecological influence compared to the primary variable (July temperature) presents special challenges to palaeo-reconstructions. We test the pollen–climate models using a novel and comprehensive cross-validation approach, running a series of *h*-block cross-validations using *h* values of 100–1500 km. Our study illustrates major benefits of this variable *h*-block cross-validation scheme, as the effect of spatial autocorrelation is minimized, while the cross-validations with increasing *h* values can reveal instabilities in the calibration model and approximate challenges faced in palaeo-reconstructions with poor modern analogues. We achieve well-performing calibration models for both primary and secondary climate variables, with boosted regression trees providing the overall most robust performance, while the palaeoclimate reconstructions from fossil datasets show major independent features for the primary and secondary variables. Our results suggest that with careful variable selection and consideration of ecological processes, robust reconstruction of both primary and secondary climate variables is possible.

## Introduction

Microfossil data (pollen, diatoms, foraminifera, chironomids, testate amoebae, ostracods) are widely employed as proxy indicators of past environmental variations, with applications in palaeoclimatology, environmental monitoring, and the study of ecosystem sensitivity, resilience and anthropogenic impact. Since the 1970s, a range of quantitative approaches have emerged to infer palaeoenvironmental variables from microfossil assemblages^1–3^. Numerical palaeoenvironmental reconstructions are generally based on a modern calibration dataset, consisting of surface sediment (i.e., chronologically recent) samples of species assemblages, with modern environmental data attached to each surface sample. Palaeoenvironmental reconstructions are then prepared using a model trained with the calibration dataset and applied to samples of fossil assemblages. A persistent challenge, however, has been to validate these reconstructions, due to limited independent knowledge about past environmental conditions^4^. Cross-validation (CV) analyses of the modern calibration data help give an estimate of reconstruction ability for fossil samples^5–8^. In this work, we explore the ability of a range of quantitative algorithms to robustly reconstruct climate variables from fossil pollen data. Our particular focus is on the reconstruction of *secondary* climate variables, i.e., variables with a lesser ecological effect on the studied biological proxy, compared to the larger effect of the *primary* climate variable^9,10^.

Several factors motivate this focus on refining methods to extract reconstructions of secondary climate variables. First, secondary variables have a long history of use in palaeoclimatology, because the multivariate nature of micropalaeontological datasets permits application of multivariate statistical methods that can, in theory, independently reconstruct multiple environmental variables, with analysis of variance enabling designation of variables with primary, secondary, etc. explanatory power^11^. However, recent literature has highlighted important challenges in the reconstruction of secondary (and further) variables. These challenges include the risk of spurious reconstructions, due to the ecological model for the secondary model lacking independence from the effects of the primary variable^10,12^, the assumption that the covariance structure between environmental variables is preserved over time^13^, and the unreliability of common CV schemes in identifying models which can reconstruct secondary variables in the presence of spatially autocorrelated and ecologically significant nuisance variables^6,8^. These results have cast doubt on many published reconstructions of secondary variables. Yet, secondary variables are important for palaeoclimatology, because they reveal diagnostic signals of the past variability in vital climate factors, forcings, feedbacks, or atmospheric-oceanic circulation mechanisms, that would otherwise be undetectable. For example, while in northern latitudes climate reconstructions from pollen and chironomids usually indicate summer temperature as a primary variable, past changes in winter temperature or precipitation may be vital to detecting and understanding past variation in sea ice extent^14^, oceanic circulation^15^, or drought regimes^16,17^. In ideal situations, palaeoclimate information is available from multiple proxies, allowing an independent validation^4^ of reconstructions for hard-to-reconstruct variables such as moisture^18^ or winter temperature^19^.

Second, there is a strong basis in ecological theory for the reconstruction of secondary variables: species distributions and abundances are affected by multiple environmental variables, and each species is likely to have its own unique fundamental niche^20,21^. Taxon responses to past and present climate changes are highly individualistic^22^, and indicator taxa exist for a number of climate variables^9,23,24^. Thus some signal of multiple climate variables should be extractable from multivariate fossil datasets that include multiple indicator taxa^25^, though potentially challenging in practice.

Our final motivation is the on-going and rapid advances in a relatively new class of reconstruction approaches, consisting of machine-learning (ML) algorithms that use ensemble models of regression trees^7,9,26,27^. ML approaches have important theoretical strengths in the reconstruction of secondary variables. First, tree ensembles make selective use of predictor variables, and are thus able to focus on the potentially small number of useful indicator taxa for a secondary variable, while ignoring the numerous and abundant indicators of the primary variable. Second, the tree models give equal weight to rare and abundant taxa, which can help capture the signal of secondary variables in the variation of relatively rare fossil taxa^7,27,28^. Beyond these conceptual strengths, an increasing number of recent studies suggest regression tree ensembles can provide well-performing calibration models for microfossil proxies^7,9,26,27,29^ and for species distribution models applied to fossil pollen data^30^. Here, we employ three types of regression tree ensemble models: random forest (RF), boosted regression tree (BRT), and extremely randomized trees (ETREES). We also test another family of ML models, artificial neural networks, using both the traditional (NNET) and the Extreme Learning Machine (ELM) implementations. This is the first use of two of these ML methods (ETREES, ELM) in palaeoclimate reconstruction.

**Figure 1:**
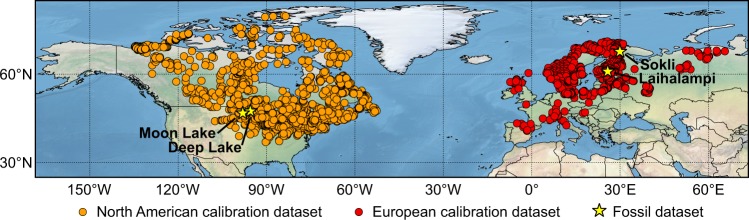
Modern and fossil pollen datasets in North America and Europe.

In this study, we test the ability of pollen–climate calibration models to detect the signals of both primary and secondary climate variables, while controlling for two key sources of bias. First, we select minimally correlated primary and secondary variables, to ensure the independence of the pollen–climate calibration models. Second, we evaluate the models using a new CV approach, a variation on *h*-block CV^6,8^ that employs a variable radius. In this approach, the models are tested in several CV cycles while omitting samples from an increasingly large radius (*h*) around the test sample. In this way, model performance is evaluated with increasingly poor analogues available for the test sample in the calibration data, providing a strong test of the generality and robustness of the models. This approach also allows *h* to be customized to the dataset at hand, to find the optimal tradeoff between removing spatial autocorrelation effects and maximizing calibration dataset size. We prepare the models using pollen–climate calibration datasets (surface sediment pollen samples and modern climate data) from North America and Europe (Fig. 1). Finally, we test these models in palaeo-reconstructions using four fossil pollen datasets of the present (Holocene) and last interglacial (LIG) periods from North America and Europe (Fig. 1, Table 1). We use eight quantitative methods to prepare the calibration models (Table 2), with the five ML-based methods compared against three classical approaches (weighted averaging (WA), weighted averaging-partial least squares (WAPLS), and the modern analogue technique (MAT)). We show that the pollen–climate calibration models developed here perform well for both primary and secondary variables. Further, we find regression-tree-based ML approaches to consistently outperform other reconstruction methods.

**Table 1:** Fossil pollen datasets used for the study.

Site	# Samples	Time range (cal. ka)
Deep Lake, Minnesota^73,74^	62	0.2–11.2
Moon Lake, North Dakota^75^	170	0–14.0
Laihalampi, Finland^76^	150	0–11.0
Sokli, Finland^19,41^	217	117.4–130.3

**Table 2:** Modelling tools used and their parameterization.

Code	Method	Parameters
MAT	Modern analogue technique	Weighted mean of 5 closest analogues
WA	Weighted averaging	Monotonic deshrinking, tolerance down-weighting, square-root transformation of species data
WAPLS	Weighted averaging-partial least squares	3-component models, square-root transformation of species data
RF	Random forest	100 trees
ETREES	Extremely Randomized Trees	Number of random cuts = 5
BRT	Boosted regression trees	Maximum number of trees = 3000, learning rate = 0.025, tree complexity = 4, bagging fraction = 0.5
NNET	Artificial neural network	Linear output units. Number of units in the hidden layer = 18 (Europe, *T*_jul_), 19 (Europe, *T*_jan_), 13 (North America, *T*_jul_), or 8 (North America, Water balance)
ELM	Extreme Learning Machine	Prediction with a mean of 5 networks. Positive linear activation function. Number of units in the hidden layer = 180 (Europe, *T*_jul_), 130 (Europe, *T*_jan_), 290 (North America, *T*_jul_), or 280 (North America, Water balance)

## Results

### Variable selection

**Figure 2:**
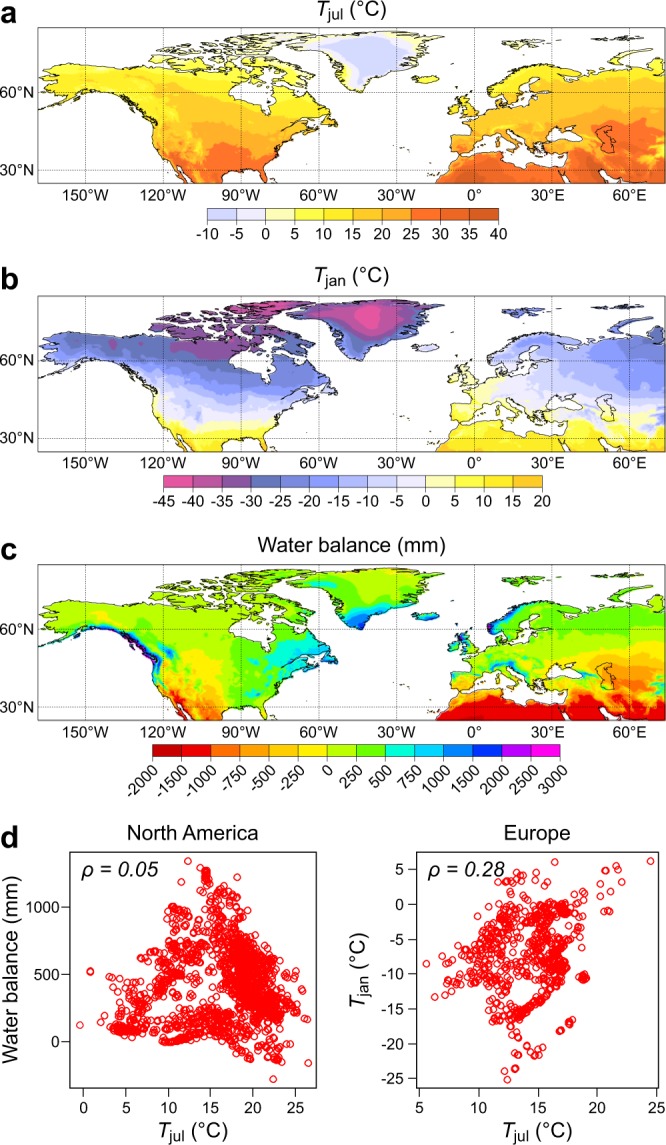
Spatial distribution and correlation of reconstructed variables. The maps show the modern values for (**a**) July mean air temperature (*T*_jul_), (**b**) January mean air temperature (*T*_jan_), and (**c**) Annual water balance. Panel (**d**) shows observed values and the Spearman correlation (*ρ*) for the reconstructed climate variables for the European and North American pollen–climate calibration datasets.

We choose mean July temperature (*T*_jul_; Fig. 2a) as the primary reconstructed variable for both North America and Europe, due to the strong effect of this variable on calibration species data variation. The secondary reconstructed variables used differ between North America and Europe, due to the different cross-correlation structures between climate variables on the two continents. Most notably, in North America summer and winter temperature related variables are strongly correlated, while in Europe they are not. We thus select mean January temperature (*T*_jan_; Fig. 2b) as the secondary variable to be reconstructed in Europe, however in North America water balance (Fig. 2c), i.e. the difference between the total annual precipitation and evapotranspiration^24^, is reconstructed. These secondary variables are known to be ecologically important with respect to affecting plant distributions and abundances and each has a low correlation with the primary reconstructed variable *T*_jul_ (Fig. 2d).

### Cross-validations

**Figure 3:**
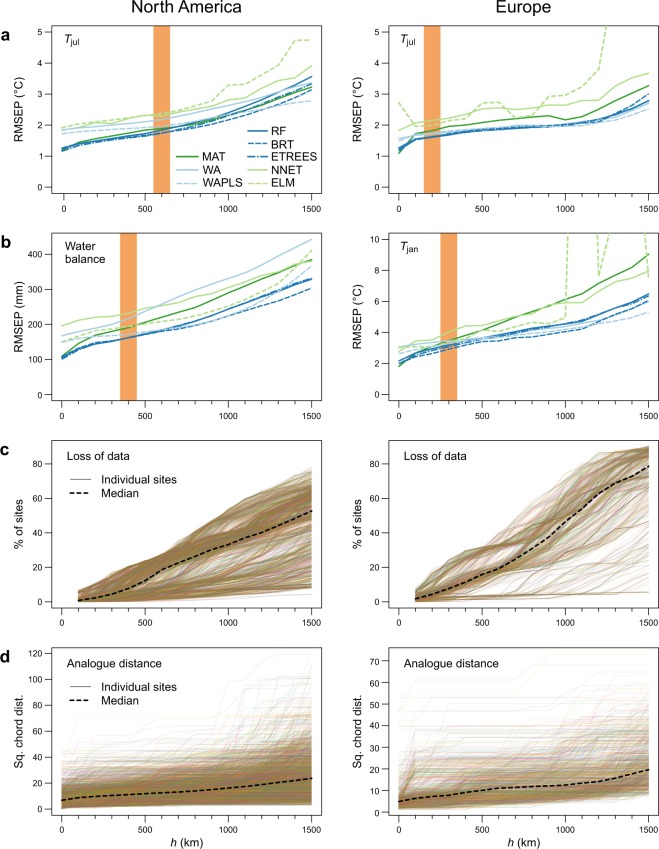
Cross-validation (CV) results. Results are shown with a series of *h-*block CV’s, with *h* increasing from 0 to 1500 km at 100 km increments (note: *h* = 0 is equivalent to leave-one-out CV). *Orange bars* indicate the best *h*, estimated based on the range of a variogram fitted to the residuals of a weighted averaging (WA) model. (**a**) Root-mean-square error of prediction (RMSEP) for the primary variable in North America and Europe (July mean temperature). (**b**) RMSEP for the secondary variable (water balance in North America, January mean temperature in Europe). (**c**) Loss of calibration data in CV models, shown for individual sites and as the median for all sites. (**d**) Compositional distance (squared chord distance) to best pollen analogue in CV models, shown for individual sites and as the median for all sites.

Transitioning from leave-one-out CV (*h* = 0) to a small *h* of 100–200 km produces an initial decrease in model performance, indicated by an increase in RMSEP (Fig. 3a,b). This is expected from earlier *h*-block experiments^6–8,26^, due to the removal of pseudo-replicate samples that closely resemble the test sample due to spatial autocorrelation in nuisance variables. This initial decrease in performance varies between methods and is especially noteworthy for MAT in Europe, where MAT is the best-performing method in leave-one-out CV, but falls behind the best-performing methods with increasing *h* (Fig. 3a,b). At intermediate *h* values (~200–1000 km), prediction performance decreases only gradually, and the relative performance of the methods remains largely unchanged. At large *h* values (>~1000 km) model performance markedly deteriorates (Fig. 3a,b), as the number (Fig. 3c) and quality (Fig. 3d) of available analogues in the calibration data worsens. As at small *h* values, this deterioration is larger for some methods, particularly MAT and neural networks (NNET, ELM), compared to unimodal transfer functions (WA, WA-PLS) and regression tree ensembles (RF, BRT, ETREES) which perform better in prediction with poor analogues.

This intermediate *h* range, and the corresponding RMSEP-vs-*h* plateau is expected to give an unbiased estimate of predictive ability, with the effect of pseudoreplicate samples minimized, but with the models still having sufficient data for prediction^7^. We independently estimated the correct *h* to use based on the range of a circular variogram fitted to the residuals of a WA model (as suggested in refs^6,8^). These estimates for correct *h* (200–600 km; orange bars in Fig. 3) fall on the observed RMSEP-vs-*h* plateaus (Fig. 3a,b), indicating a congruence among methods.

In the medium-*h* zone expected to give unbiased estimates, the tree-ensemble approaches (BRT, ETREES, RF) have the best performance and rank as the top three methods in all cases (Table 3). Among these three methods, BRT has the lowest RMSEP in three cases out of four, while ETREES has the lowest RMSEP for *T*_jul_ in North America. While the RMSEP differences between the tree-ensemble methods are relatively minor, in the European models for both *T*_jul_ and *T*_jan_, BRT has a lower maximum bias by a considerable margin compared to ETREES and RF (Table 3). The three tree-ensemble methods are followed by WA, WAPLS, MAT and ELM in varying order, while NNET is the worst-performing method, ranking at bottom in three cases out of four and second-worst in the fourth case. The overall performance achieved is strong, with the RMSEP of the best-performing model, for each dataset-variable pairing, constituting between 6.5% (*T*_jul_ in North America) and 10.0% (water balance in North America) of calibration data gradient length. The coefficients of determination (*R*^2^) of the models range from 0.20 (NNET model for North American water balance) to 0.88 (ETREES model for North American *T*_jul_). For further details, see Supplementary Figs S5–S8.

**Table 3:** Cross-validated performance metrics for the individual pollen–climate calibration models.

Rank	North America, *T*_jul_ (°C)			North America, Water balance (mm)			Europe, *T*_jul_ (°C)			Europe, *T*_jan_ (°C)		
	Method	RMSEP	Max.bias	Method	RMSEP	Max.bias	Method	RMSEP	Max.bias	Method	RMSEP	Max.bias
1	ETREES	1.73	6.87	BRT	161.55	529.50	BRT	1.61	5.59	BRT	2.92	7.16
2	BRT	1.75	6.91	RF	161.92	570.55	RF	1.61	7.75	ETREES	3.08	9.36
3	RF	1.84	7.92	ETREES	162.14	557.45	ETREES	1.63	7.81	RF	3.19	9.66
4	MAT	1.89	5.14	WAPLS	171.97	595.71	WA	1.72	7.47	WAPLS	3.28	10.29
5	WAPLS	1.96	7.88	MAT	190.71	574.10	WAPLS	1.73	5.90	ELM	3.37	10.00
6	WA	2.18	8.71	ELM	194.05	612.41	MAT	1.82	8.78	WA	3.46	13.48
7	NNET	2.29	7.54	WA	216.10	676.09	ELM	1.98	6.35	MAT	3.47	6.73
8	ELM	2.37	6.83	NNET	231.63	660.83	NNET	2.15	7.77	NNET	4.03	7.99

Results are shown for eight models and for primary and secondary climate variables in each calibration dataset (North America and Europe), using *h-*block cross-validation with *h* determined by the variogram-range method. The metrics shown for each model are root-mean-square error of prediction (RMSEP) and maximum (Max.) bias. Models are ranked based on increasing RMSEP.

### Palaeo-reconstructions

**Figure 4:**
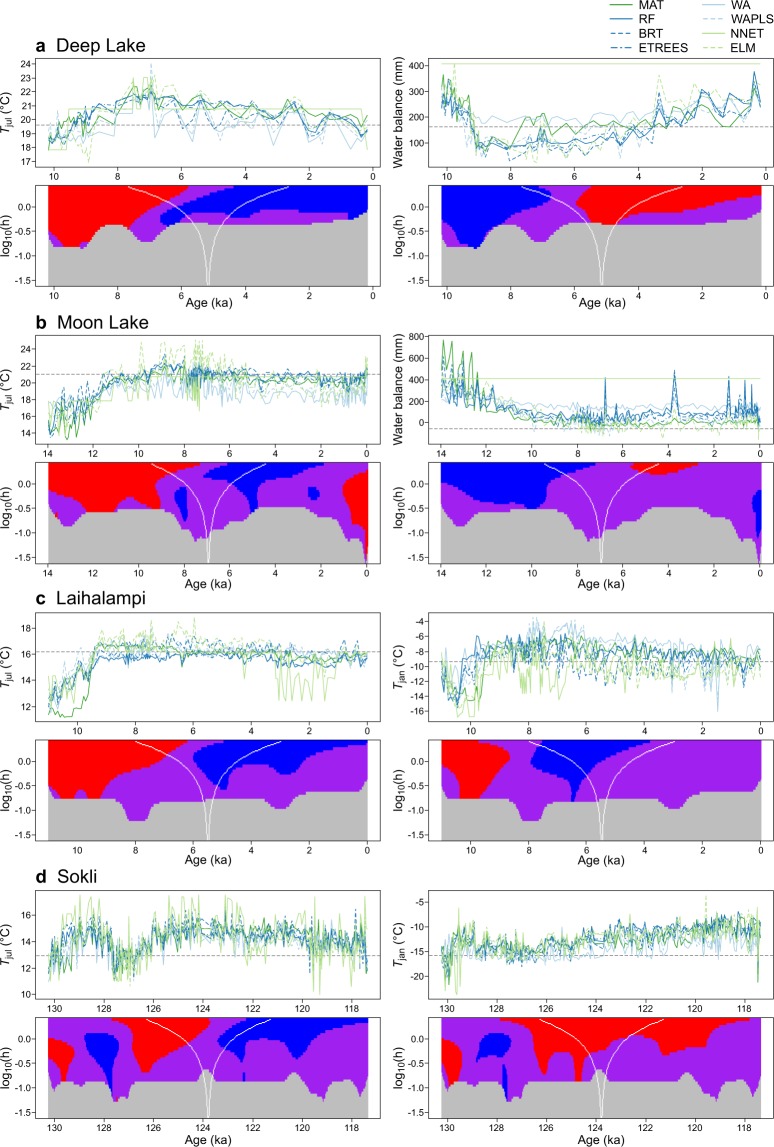
Palaeoclimate reconstructions. Reconstructions are shown for primary and secondary climate variables and prepared with eight calibration methods from each fossil dataset. The black dashed lines indicate the modern climate values at the fossil sites. The SiZer maps (lower panels) show the significant features of the reconstructions, based on the curve using the calibration method with the strongest CV performance for the climate variable in question (BRT or ETREES; Table 3). The reconstruction is smoothed at different bandwidths, with the bandwidth used at each point on the vertical axis indicated by the horizontal distance between the white lines. For each point in time and each bandwidth (*h*), *red* indicates a significant rising trend, *blue* a significant falling trend, *purple* a lack of a significant trend, and *grey* a lack of sufficient data for meaningful inference.

In the reconstructions of Holocene climate variations at the two North American test sites (Fig. 4a,b), the main feature in *T*_jul_ is the early-Holocene rise, followed by a late-Holocene fall, showing the well-known mid-Holocene temperature maximum of the northern mid-latitudes^31^. The water balance curves, by contrast, show an early-Holocene decline, culminating in a dry period starting around 8 ka (depending on smoothing bandwidth considered), and followed by a gradual rise in water balance at around 6 ka. The early–mid Holocene maximum in aridity is consistent with numerous multi-proxy records from the Great Plains^17,18,32–34^. The strong differences in temporal pattern further suggests that both temperature and water balance signals can be separately deconvolved from mid-continental North American pollen records^16,18,32,34^.

The European Holocene reconstructions (Fig. 4c) for *T*_jul_ and *T*_jan_ are broadly similar to each other. In the long-bandwidth (>1 ka) end of the SiZer map, the early-Holocene rise, mid-Holocene maximum, and late-Holocene decline are statistically significant features for both variables. The *T*_jul_ reconstruction thus shows the classical, mid-Holocene summer temperature maximum of the European high latitudes seen in palaeoclimate reconstructions^35,36^ and modelling^37^. The *T*_jan_ reconstruction is more difficult to validate compared to *T*_jul_, because of broader uncertainty about whether the temporal variations of *T*_jan_ and *T*_jul_ should be correlated (e.g. due to GHG forcing) or anti-correlated (e.g. due to orbital forcing)^31,38,39^. Earlier reconstructions^35,36^ and modelling^37^ have shown only weak trends for *T*_jan_ in NE Europe, and reconstruction and modelling uncertainties are much greater for *T*_jan_ compared to *T*_jul_. However, our mid-Holocene maximum in *T*_jan_ is consistent with fossil evidence for one well-understood winter temperature indicator in Northern Europe, the hazel (*Corylus avellana*), which shows a major, northward range expansion in Finland and Scandinavia during the mid-Holocene^40^.

By contrast, for the European LIG site (Fig. 4d), the main features at the long-bandwidth end of the SiZer map differ between *T*_jul_ and *T*_jan_. The *T*_jul_ reconstruction shows an early-LIG rise, mid-LIG maximum followed by a late-LIG cooling. However, the *T*_jan_ reconstruction only shows a significant warming trend spanning much of the LIG. These trends are consistent with major identified forcings, as the first-order rising (*T*_jan_) and falling (*T*_jul_) temperature trends closely follow the changes in winter and summer insolation, respectively^19^. At shorter bandwidths, the beginning and end of an abrupt cold event at ca. 128–126 ka, correlated with changes in North Atlantic circulation^19,41^, are also significant features.

The agreement between different methods is generally good in all reconstructions, however increased spread is observed especially in early parts of the interglacials and the late-glacial section included in the Moon Lake record (Fig. 4d). The periods of larger spread between methods generally coincide with periods of increased modern analogue distances found for the fossil samples (Supplementary Fig. S9), likely due to non-climatic effects during pioneer vegetation stages or non-analogue climates not included in the calibration data^42^. However, NNET is in several instances a clear outlier. For the European fossil sequences (Fig. 4c,d), the NNET curves exhibit larger sample-to-sample noise compared to other methods, and for some sections of the Laihalampi sequence (Fig. 3c) also indicate much colder temperatures than other methods. In North America, NNET jumps between a handful of values in the *T*_jul_ reconstructions, and reconstructs no variation in water balance but only a single outlier value. For water balance in North America, a second outlier is seen in WA, for which the mid-Holocene decline is considerably smaller than for the multi-method median. The reason for the shallower water balance anomaly with WA might be the well-known weakness of this method in predicting for samples near the ends of the calibration-data gradient^4,43^. This tendency is evident in the residual pattern of the WA-based pollen–water balance model, which (along with NNET) has a considerably larger positive bias among all eight models at the dry end of the modern water balance gradient (Supplementary Fig. S6), likely contributing to a positive bias in the palaeoclimate reconstruction during the mid-Holocene dry stage.

## Discussion

Based on these findings, we consider BRT or other regression tree ensemble machine-learning methods as highly promising tools for climate reconstructions from fossil pollen data. The other regression tree ensemble methods RF and ETREES show a broadly similar performance and behaviour in CV (Fig. 3) compared to BRT, and also produce similar palaeo-reconstructions (Fig. 4). The most important difference between BRT, RF, and ETREES is found in maximum bias, where BRT significantly improves on RF and ETREES in three cases out of four. The maximum bias usually affects the gradient ends^7,26^, and this is also seen in the residual patterns of the models prepared in this study (Supplementary Figs S5–S8). This means BRT is relatively strong in predicting for samples located near the ends of the environmental gradient covered by the calibration data. Hence, BRT may be particularly useful for palaeo-reconstructions in situations where the fossil dataset is located towards a fringe of the available calibration data.

Beyond the strong performance, we note that BRT has numerous practical benefits in applications with microfossil datasets. First, among these calibration methods, BRT has unusually powerful tools to analyse the model structure. For example, the user can extract the percentage contributions and plot the modelled responses curves of each taxon^9,26,44^, which can be invaluable to estimate the effect of each taxon and to verify that the models are consistent with prior ecological knowledge^19^. For example, Table 4 shows that the secondary-variable models have a distinct structure compared to the primary-variable models, and well-understood indicators for the secondary variables, such as *Corylus* and *Quercus* for *T*_jan_ in Europe and *Artemisia* and Chenopodiaceae for water balance in North America, are employed. Second, the BRT models are not affected by monotonic data transformations performed on the predictor set (here, calibration species data). Third, BRT can handle complex responses and assumes no specific response shape, which is a benefit with microfossil calibration datasets which commonly show a mixture of linear, unimodal and multi-modal species responses. This is in contrast with parametric calibration methods assuming a specific response type, such as WA and WA-PLS which fit unimodal response functions regardless of the shape of the underlying response. Fourth, BRT implicitly incorporates interactions between predictors, e.g., situations where a given taxon is only useful in a subset of the calibration data, or indicates different environmental conditions in different subsets^44^.

**Table 4:** Relative contribution of the ten most important predictor taxa for the boosted regression tree pollen–climate models for July mean temperature (*T*_jul_), January mean temperature (*T*_jan_), and water balance.

Rank	North America, *T*_jul_		North America, Water balance		Europe, *T*_jul_		Europe, *T*_jan_	
	Taxon	%	Taxon	%	Taxon	%	Taxon	%
1	*Quercus*	53.5	*Abies*	29.7	*Selaginella*	20.3	*Quercus*	17.3
2	Cyperaceae	9.9	*Pinus*	12.6	Cyperaceae	7.9	*Picea*	13.4
3	*Picea*	6.0	*Artemisia*	8.8	*Quercus*	6.9	*Betula*	12.3
4	*Salix*	4.2	Chenopodiaceae	4.5	*Juniperus*	6.0	*Corylus*	11.9
5	Chenopodiaceae	4.0	*Salix*	4.5	*Alnus*	5.3	Poaceae	8.2
6	*Pinus*	3.5	Lycopodiaceae	4.5	*Artemisia*	5.1	*Pinus*	3.7
7	*Betula*	2.4	*Picea*	3.9	Polypodiaceae	4.2	*Rumex*/*Oxyria*	3.5
8	*Ulmus*	1.7	*Betula*	3.7	*Betula*	3.8	Ericaceae	2.9
9	*Oxyria*	1.7	*Quercus*	2.6	Ericaceae	3.3	Polypodiaceae	2.2
10	Ericaceae	1.6	*Fagus*	2.3	Chenopodiaceae	3.2	*Juniperus*	2.0

However, BRT may not be the optimal choice with small datasets (*n* < 100) and/or data exhibiting strong linear responses^45^, a limitation also observed with relatively small microfossil proxy calibration datasets^7^. A further practical challenge of BRT is the non-trivial parameterization (Table 2), which requires CV testing with alternative parameterizations for each dataset^44^. Moreover, BRT uses relatively large ensemble models (ca. 2,000–20,000 trees) which can be a challenge especially with large datasets and in calculation-intensive CV schemes like the leave-one-out or the *h*-block, potentially involving calculation times of several CPU hours for a full CV cycle. Hence, for some applications, RF may represent a cost-effective alternative to BRT, with similar predictive performance (Fig. 3, Table 2) here reached with small ensemble models of 100 trees, and requiring no further parameterization. The practical benefits of BRT also largely apply to RF, as they arise from the general properties of regression tree based modelling^28^.

Among neural network models, we find ELM to improve on the classical NNET, which has the overall weakest performance of all methods (Table 2). In palaeo-reconstructions (Fig. 4), NNET has clear trouble producing diverse predictions for the fossil samples. For North-American water balance only a single value is reconstructed. For North-American *T*_jul_ the reconstructions are semi-discrete, with most data points falling on a handful of values, although the reconstructions generally follow the main features of the reconstructions with other methods. ELM generally outperforms NNET in CV (Fig. 3, Table 3) while also producing more realistic palaeo-reconstructions (Fig. 4). However, ELM fails spectacularly during CV for some *h* values (Fig. 3), suggesting great sensitivity to small variations in the calibration data. We thus recommend caution in the use of ELM until its behaviour is better understood. However, this result showcases another benefit of the variable-radius *h-*block CV, because it reveals an instability in ELM that would have been missed with a single CV cycle.

Good CV performance is no guarantee of reconstruction ability. Even if the CV scheme used gives a robust estimate of predictive ability in the modern world, fossil samples come with additional caveats, such as non-analogue climates not represented in the modern calibration data^13,46^ and taphonomic inconsistencies between some calibration and fossil data^29^. Thus, the criteria used here (low correlation to other environmental factors, a significant effect in calibration data, an ecological basis for selection) should be considered necessary but not sufficient guarantees that a useful palaeo-reconstruction is obtained for the variable in question. Large-radius *h*-block CV may approximate the problem of no analogues, due to spatial autocorrelation in the calibration species data: as calibration data is lost with increasing *h* (Fig. 3c) the predictions are done with increasingly poor analogues available for the test sample (Fig. 3d). In our results, we see important differences in the CV performance at high *h* values (>1000 km), with especially MAT and neural network based models (NNET, ELM) struggling compared to the other approaches (Fig. 3a,b).

Our reconstructions from fossil datasets (Fig. 4) are mainly proofs of concept, in which we check the results for major suspicious features, e.g., differences between methods, high noise, or major patterns inconsistent with prior knowledge. These are possibly “easy” test cases, with e.g. exceptionally strong opposite trends in winter and summer temperature forcing during the LIG^19,37^ and prior multiproxy evidence for major mid-Holocene aridity in the Great Plains of North America^17,18,33,34,47^ as well as prior applications of pollen-based palaeoclimatic transfer functions to separately reconstruct past temperature and moisture variations^16,48,49^. Whether robust and repeatable reconstructions can be achieved for secondary or tertiary variables across a larger body of micropalaeontological data remains a question for future research. These efforts should include not only the refinement of the proxy–climate calibration models, but also an increasing use of supporting multi-proxy data to control for proxy-specific biases.

While multivariate climate reconstructions from pollen and other microfossil proxies have been prepared for decades, important pitfalls have been identified in this approach, including the possible lack of model independence^10^ and issues in model validation^6^. Here we show that reconstruction of primary and secondary climate variables is indeed possible, at least for some variables and regions, given careful variable selection, consideration of the proxy ecology, and sufficiently sensitive reconstruction algorithms. We find major independent features in our primary and secondary variable palaeo-reconstructions, including the opposite summer and winter temperature trends of the LIG in Europe, as well as the mid-Holocene drought in North America coinciding with the temperature maximum. These opposite first-order trends in primary and secondary variables emerge despite weak positive correlations in the calibration data, which means the secondary-variable reconstructions are unlikely to be driven by calibration data correlations (*sensu* ref.^10^). Considering the independent features in the palaeo-reconstructions, the agreement with the identified climate forcings and complementary proxy data, the robust CV performance, and the ecological realism of the calibration models, we suggest current advanced machine-learning techniques are able to detect the independent signals of both primary and secondary climate variables. Hence, this work supports the judicious use of numerical techniques and microfossil data to reconstruct both primary and secondary climate variables.

## Conclusions

- Well-performing pollen–climate calibration models were achieved for secondary climatic variables (January temperature in Europe, water balance in North America), despite a conservative CV scheme, and in the absence of correlations with the primary climate variable (July temperature). Palaeoclimate reconstructions prepared from fossil datasets for the primary and secondary variables show independent features consistent with known climate forcings, palaeoclimate modelling, and other proxy data.
- Among different calibration techniques, regression tree ensemble methods (BRT, RF, ETREES) generally perform best. BRT further outperforms RF and ETREES in maximum bias, particularly for samples located near the ends of the data gradient. In prediction for samples without good analogues in the training data, MAT and neural network models (NNET, ELM) perform considerably worse than the other methods.
- Our study highlights the usefulness of variable-radius *h*-block CV as a practical and a neutral scheme for calibration model selection. This approach (1) removes the effect of spatial autocorrelation from the CV results, (2) helps identify the *h* range giving unbiased performance estimates, (3) tests model behaviour with poor modern analogues at large *h* values, and (4) may reveal the instability of a calibration method with small data variations.
- Overall, these analyses show how concerns about the robustness of palaeoclimatic reconstructions due to effects of spatial autocorrelation and temporally varying cross-correlation can be allayed through use of newer ML approaches and careful attention to the CV method. Reconstruction of secondary variables is possible, at least for some regions and variables. Careful consideration of the underlying ecology of the biotic proxy being used, and of the modern cross-correlation structure of environmental variables, are vital to guarantee that useful and independent calibration models are obtained for both the primary and secondary variables.

## Methods

### Datasets

The North American modern pollen data (2254 samples) were derived from a subset of the North American Modern Pollen Database^50,51^, with samples removed if they source from regions that floristically differ from the north-central US, where the two fossil pollen sites are located. For this analysis, all samples from the southeastern US and western North America were removed, due to different species of *Pinus* and other taxa found in these regions and known interregional differences in species-climate relationships^51^. For Europe, we use an 807-sample pollen–climate calibration set derived from the European Modern Pollen Dataset^52^ (EMPD), including lakes from the northern part of the EMPD and using a harmonized taxonomy of 73 terrestrial pollen and spore types^19^. We extracted climate data for both the European and North American calibration samples from the CRU CL v. 2.0 climate grids^53^ with the *raster* library^54^ for R^55^, using bilinear interpolation based on four closest grid cells and lapse-rate corrected (6.4 °C/km) based on the difference between site elevation and grid cell elevation.

To test the pollen–climate models in palaeo-reconstruction we use four previously published fossil pollen datasets (Table 1). The North-American datasets cover the Holocene (and about 2 ka of the late-glacial period in the Moon Lake dataset) and are located at the prairie–mixed forest ecotone at the eastern fringe of the North American Great Plains. The European sites Laihalampi and Sokli are located in Finland within the boreal forest zone and cover the present and last interglacials, respectively. The North American datasets were acquired from the Neotoma Paleoecology Database. The Laihalampi dataset was provided by the original data author, while the Sokli dataset was published by the present authors^19^.

### Variable selection

To guide the selection of reconstructed climate variables, we consider the following to be requirements for a useful variable. The variable should have:

1. a significant effect on species variation in the calibration data,
2. a low correlation with other ecologically significant variables, to guarantee that the effect can be independently modelled, and by extension, that no spurious features emerge in palaeo-reconstructions^10^, and
3. an autecological basis, to guarantee that the statistical effect (point 1) is not due to correlation with unknown, ecologically significant variables.

To find the two climate variables for each region (Europe and North America) that best meet these requirements, we use the following workflow:

1. Calculate a Spearman correlation matrix for a large number of climate variables. We included 20 variables with possible ecological influence, representing temperature (*annual mean, December-to-February mean, mean of coldest month, January mean, June-to-August mean, mean of warmest month, July mean, number of frost days, growing degree days*) precipitation (*annual total, total for driest month, total for wettest month, total for warmest quarter, total for coldest quarter*) and moisture availability (*water balance* (difference between annual precipitation and evapotranspiration^24^)*, Priestley-Taylor alpha* (ratio of actual to potential annual evapotranspiration^23^)), and their seasonal variation (*temperature range* (range of monthly temperature means)*, temperature seasonality* (SD of monthly temperature means × 100)*, precipitation seasonality* (ratio of largest to smallest monthly precipitation total)*, continentality index*^56^).
2. From the full set of 20 variables, pick a subset for which *all* between-variable absolute correlations are < 0.7. (Note: at this stage the variables are not taken as ecologically meaningful, merely that their independent effects can be modelled.)
3. Run an ensemble of modelling tools to estimate how well the variation in each climate variable is explained with the calibration species data. Rank the variables based on variance explained (mean *R*^2^ across for all modelling tools used). Here, we used an ensemble of 10 modelling tools (generalized additive models, conditional inference trees, random forests, extremely randomized trees, multivariate adaptive regression splines, generalized additive models by likelihood based boosting, gradient boosting with regression trees, gradient boosting for additive models, Extreme Learning Machine neural networks, and single-hidden-layer neural networks).
4. Check that each variable has an independent effect in non-metric multidimensional scaling; if not, exclude.
5. Check that each variable is ecologically credible; if not, exclude.
6. Pick the two best remaining variables.

The full results of these analyses are presented in Supplementary Figs S1–S4 and Tables S1–S4. The choice of reconstructed climate variables differs between Europe (*T*_jul_, *T*_jan_) and North America (*T*_jul_, water balance), due to differences in cross-correlation structure among climate variables on the two continents.

In North America, summer and winter temperature related variables are strongly correlated (*ρ* = ~0.8) (Supplementary Fig. S1), precluding using both for reconstruction, and we only choose *T*_jul_ for further consideration. Within a subset of five variables with acceptable between-variable correlations (Supplementary Fig. S2), pollen–climate models for *T*_jul_ have the highest mean *R*^2^ (0.89) (Supplementary Table S1), and *T*_jul_ is thus selected as the primary variable. *T*_jul_ is followed in the *R*^2^ ranking by two moisture-related variables, precipitation of the warmest quarter (0.75) and water balance (0.71). Of these variables, we select water balance as the secondary variable despite a slightly lower *R*^2^, due to being a moisture-related variable incorporation evapotranspiration (see below), and due to having only a minimal (*ρ* = 0.05) although still statistically significant (*p* = 0.02) correlation with the primary variable *T*_jul_, while for precipitation of the warmest quarter the correlation to *T*_jul_ is considerably higher (*ρ* = 0.46).

In Europe, summer and winter temperature related variables are much lower correlated (Supplementary Fig. S3), allowing both *T*_jul_ and *T*_jan_ (correlated at *ρ* = 0.28; p < 0.001) to be considered. Within the subset of five variables with acceptable between-variable correlations (Supplementary Fig. S4), *T*_jan_ and *T*_jul_ are the two variables with highest mean *R*^2^ values (0.79 and 0.72, respectively; Supplementary Table S3), and are thus selected as the two reconstructed variables. While *T*_jan_ has a higher *R*^2^ compared to *T*_jul_ in the cross validation, we designate *T*_jul_ the primary variable as our fossil datasets are located in the northern subset of the calibration data, and here the effect of summer temperature is clearly dominant to winter temperature^9,26^, and the signal of *T*_jul_ is thus expected to be stronger in these fossil datasets.

Our three climate variables (*T*_jul_, *T*_jan_, water balance) reflect principal limitations on plant growth and survival^23,24,57^. In seasonally variable environments, annual mean temperature does not represent the growing season or over-wintering conditions, which play a more central role in governing the distribution and abundance of plants^58,59^. In high- and mid-latitudes in the northern hemisphere, *T*_jul_ describes the temperature of the warmest month and overall growing season conditions, whereas *T*_jan_ indicates the wintertime conditions and general stress (related to overwintering survival) of the coldest period of the year. Predictors representing water availability for plants are often derived from mean annual precipitation, however precipitation is a poor surrogate for plant-available water. This is because water availability is strongly related to evaporation, for example in cold climates 500 mm rainfall per year produces a positive water balance (precipitation minus evapotranspiration), whereas in temperate systems the same amount of rainfall creates semi-arid conditions with a negative water balance. Thus, water balance represents a more accurate measure of plant available water compared with precipitation^59^.

### Calibration methods

We use eight quantitative reconstruction approaches which can be divided into four methodological families. The models were run in R^55^ using parameterizations listed in Table 2. The CV runs were performed on the Taito supercluster of CSC – IT Center for Science Ltd., Espoo, Finland. For the code implementing the *h-*block CV run, see Supplementary Code.

The modern analogue technique^2^ (MAT) is a traditional non-parametric reconstruction approach with microfossil data. The method looks for *n* closest modern assemblages for each fossil sample, using a chosen compositional distance metric, and calculates the reconstructed palaeoclimate value as the mean (or weighted mean) of the values at the modern sample sites. Weighted averaging^3^ (WA) and weighted averaging-partial least squares^43^ (WAPLS) are closely related methods with a long history of use in microfossil-based palaeoclimate reconstructions. They are based on fitting unimodal response functions to the modern distribution of each taxon, and then calculating the palaeoclimate value based on the modern responses of all the taxa found in the fossil sample. MAT, WA, and WAPLS were implemented with the R package *rioja*^60^.

We also use two families of machine-learning based modelling approaches: regression tree ensembles and neural networks. The random forest^61^ (RF; implemented with the R package *randomForest*^62^) and extremely randomized trees^63^ (ETREES; for implementation see ref.^64^) are ensemble models of regression trees, in which a number of trees are calculated and the final prediction calculated as the mean of the predictions from the individual trees. RF and ETREES differ in the approaches used to create variety in the individual trees: while RF uses bootstrap samples of the entire training data for each tree, and uses a randomly selected subset of the entire predictor set when determining each tree split, ETREES selects the cut point at random. Our final tree-ensemble method, the boosted regression tree^44,65^ (BRT; implemented with the R package *gbm*^66^), differs in that the ensemble is built sequentially, with each added tree aiming to explain the residuals of the previously fitted ensemble. The BRT can thus be likened to an additive regression model in which the individual terms are regression trees. RF and BRT have seen some recent use with microfossil data^7,9,19,26,27,29,30^. To our knowledge, this is the first application of ETREES in this field. Finally, we use two variations neural network algorithms, the traditional implementation^67^ (NNET) and Extreme Learning Machine^68^ (ELM). The R packages *nnet*^69^ (NNET) and *elmNN*^70^ (ELM) both implement single-hidden-layer feedforward neural networks. NNET uses sigmoid activation functions in the hidden layer and optimizes the weights of all connections. ELM has a wider variety of alternative activation functions and uses random weights in the hidden layer. NNET has seen some use in palaeo-environmental reconstructions from microfossil proxies^4^ while ELM has not.

### Cross-validation

A recognized limitation of many commonly used CV schemes is their susceptibility to over-estimate predictive ability in the presence of spatial autocorrelation in the calibration data^6,8^. The *h*-block CV^6^ has been suggested as a solution, in which in each CV iteration the training set omits not only the test sample, but also all samples within a specified radius (*h*) from the test sample. However, the *h*-block CV introduces a new challenge of choosing a correct *h* which removes pseudo-replicate samples but does not undermine performance by removing too much data^8^. Here, we adopt the approach of running *h*-block CV with a range of *h* (0–1500 km at 100 km increments). Of these, the *h* = 0 iteration is equivalent to the common leave-one-out CV. We observe the change in RMSEP with increasing *h* to estimate an *h* that represents a balance between removing pseudo-replicates but retaining sufficient data coverage^7^. As a guide to assessing the results, we also estimate the optimal *h* following ref.^6^, who suggest estimating *h* as the range of a circular variogram fitted to the residuals of a WA model in a leave-one-out CV. CV performance is summarized with 1) the root-mean-square error of prediction (RMSEP) and 2) maximum bias (the largest mean of prediction residuals found for any of the 10 equal length segments of the calibration data climate gradient), representing a “worst case” error for some segment of the calibration data climatic gradient.

### Palaeoclimate reconstructions

Palaeoclimate reconstructions for the primary and secondary variable were prepared from the fossil datasets with each of the eight calibration models. To assess the presence of independent features in the reconstructions, we prepare SiZer maps^71^ (implemented with the R package *SiZer*^72^) based on the palaeoclimate curves. Here we use the palaeoclimate curve prepared with the method which showed the strongest CV performance in predicting the reconstructed climate variable on that continent (BRT or ETREES; Table 3). In the SiZer analysis, a family of smoothers with a range of bandwidths is first applied to the palaeoclimate curve. The derivative of the smoother of each bandwidth is then analysed for significant deviations from zero, to identify time segments at which the smoother has a statistically significant rising trend, a falling trend, or no trend. The results are displayed as a two-dimensional coloured raster (the SiZer map) where the y axis indicates the smoothing bandwidth considered, x axis the point in time, and the cell colour the presence of a significant rising trend (*red*), a significant falling trend (*blue*), the lack of a significant trend (*purple*), or the lack of data for meaningful inference (*grey*).

## Supplementary information

Supplementary information

Supplementary Data file

Supplementary Code file

## Data Availability

Data (10.6084/m9.figshare.9938375) and code (10.6084/m9.figshare.8082221) related to this paper are available online.
