# Supplementary material for: Machine-learning based reconstructions of primary and secondary climate variables from North American and European fossil pollen data
Source: Sci Rep. 2019 Nov 1;9:15805. doi: 10.1038/s41598-019-52293-4 (PMC6825136; doi:10.1038/s41598-019-52293-4)
Supplement: Supplementary file 1 — Supplementary information [file 41598_2019_52293_MOESM1_ESM.pdf]

Supplementary Information

Machine-learning based reconstructions of primary and secondary climate variables from North American and European fossil pollen data

J. Sakari Salonen, Mikko Korpela, John W. Williams, Miska Luoto

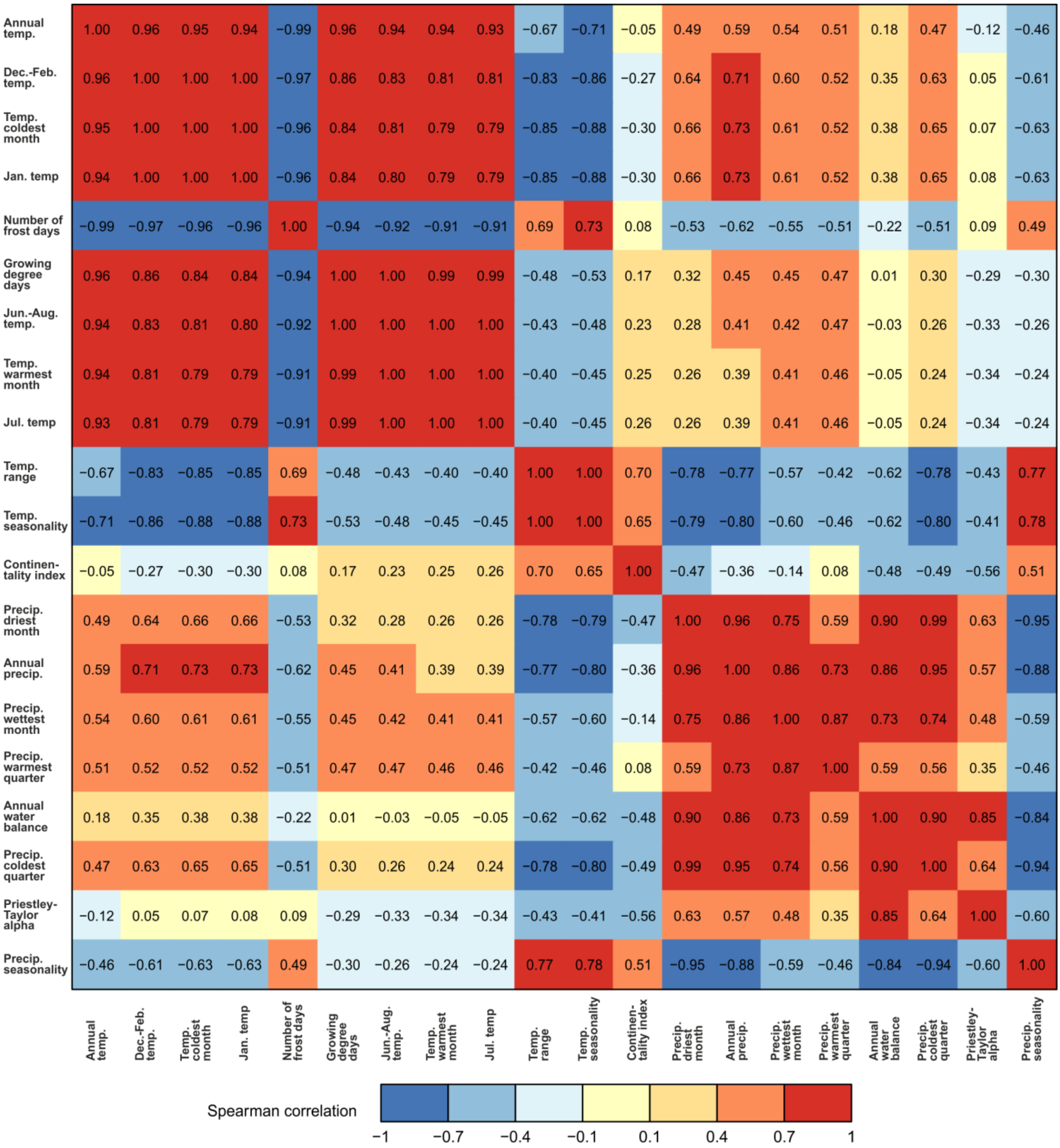

Supplementary Figure S1. Spearman correlation matrix for 20 climate variables extracted for the North American pollen calibration samples.

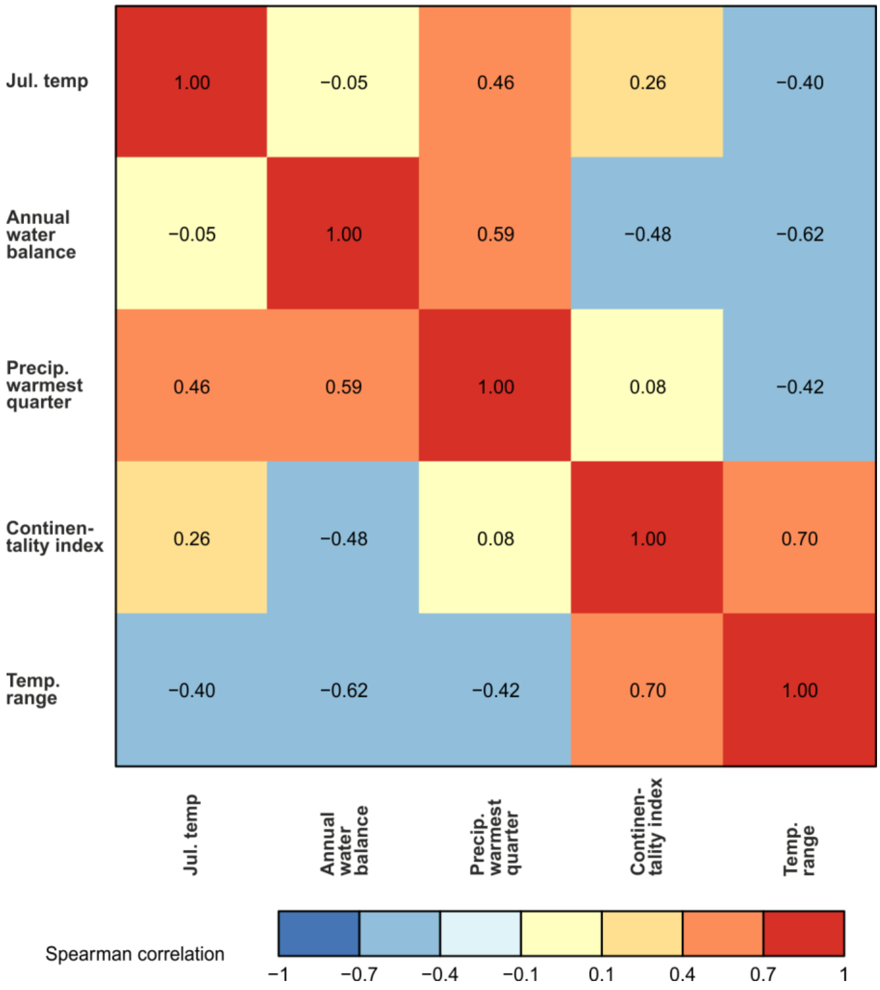

Supplementary Figure S2. Spearman correlation matrix for a subset of climate variables showing acceptable (absolute $\sigma < 0.7$) correlations in the North American pollen calibration samples.

Supplementary Table S1. Coefficients of determination (R^2) of North American pollen–climate models prepared for five selected climate variables with ten statistical modelling algorithms. The climate variables are ranked based on the highest mean R^2 value across all ten models. The R^2 values were estimated using leave-one-out (GAM, CTREE, RF, ETREES, EARTH) or 10-fold (GAMBOOST, MBLACK, MGAM, ELM, NNET) cross-validation. The modelling tools used are generalized additive models (GAM) from R CRAN package "mgcv", conditional inference trees (CTREE) from "party", random forests (RF) from "randomForest", extremely randomized trees (ETREES) from "extraTrees", multivariate adaptive regression splines from "earth" (EARTH), generalized additive models from "GAMBoost" (GAMBOOST), gradient boosting with regression trees from "mboost" (MBLACK), gradient boosting for additive models from "mboost" (MGAM), extreme learning machine (ELM) neural network from "elmNN" (archived CRAN package), and neural network (NNET) from "nnet".

Rank	Variable	GAM	CTREE	RF	ETREES	EARTH	GAMBOOST	MBLACK	MGAM	ELM	NNET	Mean
1	**July temp.**	0.88	0.88	0.94	0.94	0.86	0.88	0.91	0.87	0.89	0.88	**0.89**
2	**Precip. warmest quarter**	0.75	0.72	0.87	0.88	0.74	0.75	0.80	0.71	0.74	0.54	**0.75**
3	**Annual water balance**	0.72	0.70	0.84	0.85	0.72	0.72	0.75	0.68	0.71	0.42	**0.71**
4	**Annual temp. range**	0.67	0.62	0.81	0.84	0.60	0.66	0.75	0.60	0.63	0.61	**0.68**
5	**Continentality index**	0.57	0.50	0.73	0.76	0.52	0.56	0.66	0.47	0.57	0.40	**0.57**

Supplementary Table S2. North American Nonmetric Multi-Dimensional Scaling (NMDS) results. Pollen samples from each site were reduced to two dimensions using metaMDS (monoMDS) from R CRAN package "vegan". Five selected climate variables were fitted with vegan::envfit to the NMDS result so that the correlation between each climate variable and the ordination is maximized in the direction of vector (NMDS1, NMDS2). The climate variables are ranked based on the highest R^2 (squared correlation).

Rank	Variable	NMDS1	NMDS2	R^2
1	**July temp.**	0.98	-0.20	0.68
2	**Precip. warmest quarter**	0.68	-0.74	0.52
3	**Annual water balance**	0.10	-0.99	0.36
4	**Annual temp. range**	-0.93	0.37	0.34
5	**Continentality index**	0.84	0.54	0.0081

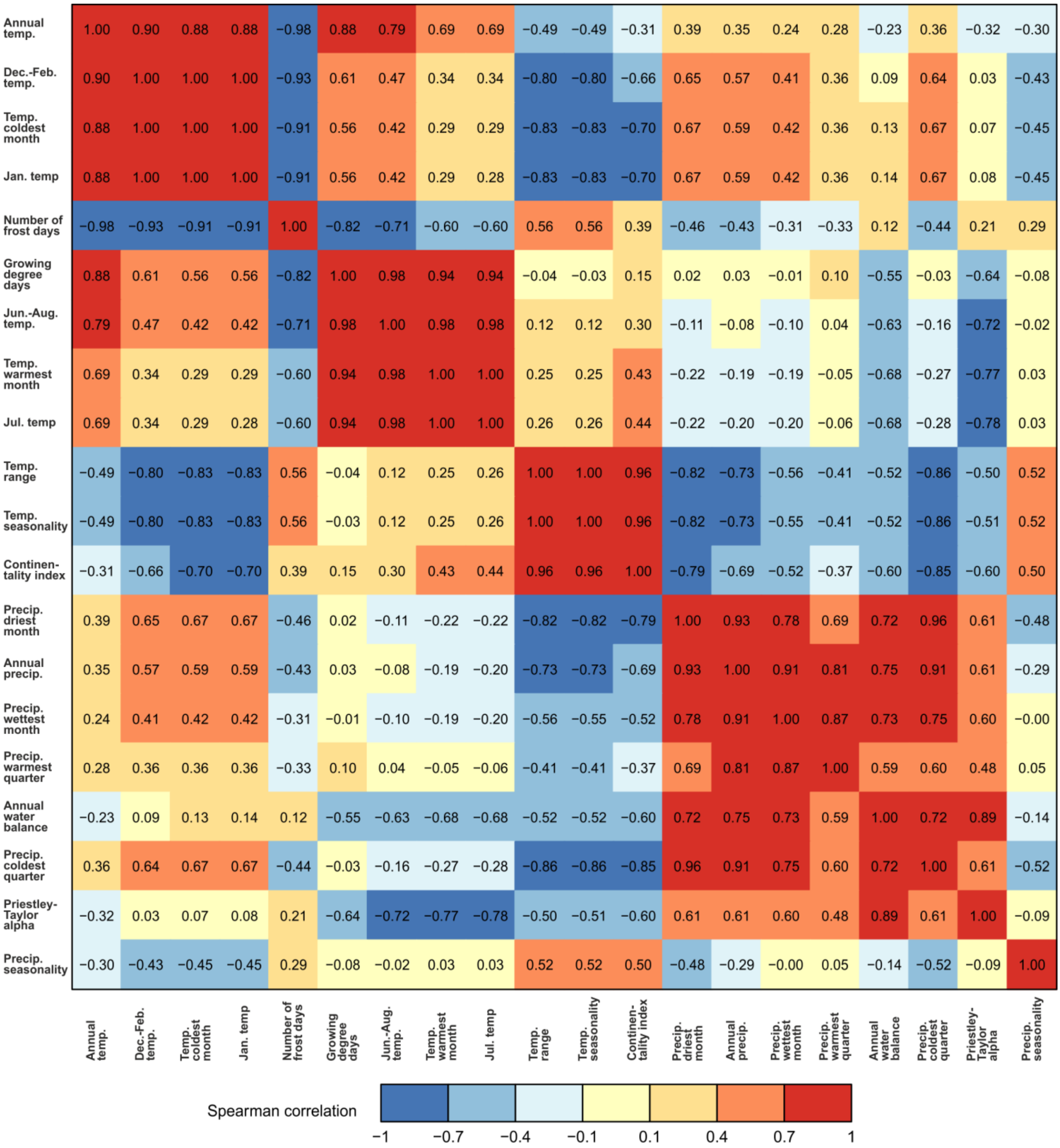

	Annual temp.	Dec.-Feb. temp.	Temp. coldest month	Jan. temp	Number of frost days	Growing degree days	Jun.-Aug. temp.	Temp. warmest month	Jul. temp	Temp. range	Temp. seasonality	Continen-tality index	Precip. driest month	Annual precip.	Precip. wettest month	Precip. warmest quarter	Annual water balance	Precip. coldest quarter	Priestley-Taylor alpha	Precip. seasonality
Annual temp.	1.00	0.90	0.88	0.88	−0.98	0.88	0.79	0.69	0.69	−0.49	−0.49	−0.31	0.39	0.35	0.24	0.28	−0.23	0.36	−0.32	−0.30
Dec.-Feb. temp.	0.90	1.00	1.00	1.00	−0.93	0.61	0.47	0.34	0.34	−0.80	−0.80	−0.66	0.65	0.57	0.41	0.36	0.09	0.64	0.03	−0.43
Temp. coldest month	0.88	1.00	1.00	1.00	−0.91	0.56	0.42	0.29	0.29	−0.83	−0.83	−0.70	0.67	0.59	0.42	0.36	0.13	0.67	0.07	−0.45
Jan. temp	0.88	1.00	1.00	1.00	−0.91	0.56	0.42	0.29	0.28	−0.83	−0.83	−0.70	0.67	0.59	0.42	0.36	0.14	0.67	0.08	−0.45
Number of frost days	−0.98	−0.93	−0.91	−0.91	1.00	−0.82	−0.71	−0.60	−0.60	0.56	0.56	0.39	−0.46	−0.43	−0.31	−0.33	0.12	−0.44	0.21	0.29
Growing degree days	0.88	0.61	0.56	0.56	−0.82	1.00	0.98	0.94	0.94	−0.04	−0.03	0.15	0.02	0.03	−0.01	0.10	−0.55	−0.03	−0.64	−0.08
Jun.-Aug. temp.	0.79	0.47	0.42	0.42	−0.71	0.98	1.00	0.98	0.98	0.12	0.12	0.30	−0.11	−0.08	−0.10	0.04	−0.63	−0.16	−0.72	−0.02
Temp. warmest month	0.69	0.34	0.29	0.29	−0.60	0.94	0.98	1.00	1.00	0.25	0.25	0.43	−0.22	−0.19	−0.19	−0.05	−0.68	−0.27	−0.77	0.03
Jul. temp	0.69	0.34	0.29	0.28	−0.60	0.94	0.98	1.00	1.00	0.26	0.26	0.44	−0.22	−0.20	−0.20	−0.06	−0.68	−0.28	−0.78	0.03
Temp. range	−0.49	−0.80	−0.83	−0.83	0.56	−0.04	0.12	0.25	0.26	1.00	1.00	0.96	−0.82	−0.73	−0.56	−0.41	−0.52	−0.86	−0.50	0.52
Temp. seasonality	−0.49	−0.80	−0.83	−0.83	0.56	−0.03	0.12	0.25	0.26	1.00	1.00	0.96	−0.82	−0.73	−0.55	−0.41	−0.52	−0.86	−0.51	0.52
Continen-tality index	−0.31	−0.66	−0.70	−0.70	0.39	0.15	0.30	0.43	0.44	0.96	0.96	1.00	−0.79	−0.69	−0.52	−0.37	−0.60	−0.85	−0.60	0.50
Precip. driest month	0.39	0.65	0.67	0.67	−0.46	0.02	−0.11	−0.22	−0.22	−0.82	−0.82	−0.79	1.00	0.93	0.78	0.69	0.72	0.96	0.61	−0.48
Annual precip.	0.35	0.57	0.59	0.59	−0.43	0.03	−0.08	−0.19	−0.20	−0.73	−0.73	−0.69	0.93	1.00	0.91	0.81	0.75	0.91	0.61	−0.29
Precip. wettest month	0.24	0.41	0.42	0.42	−0.31	−0.01	−0.10	−0.19	−0.20	−0.56	−0.55	−0.52	0.78	0.91	1.00	0.87	0.73	0.75	0.60	−0.00
Precip. warmest quarter	0.28	0.36	0.36	0.36	−0.33	0.10	0.04	−0.05	−0.06	−0.41	−0.41	−0.37	0.69	0.81	0.87	1.00	0.59	0.60	0.48	0.05
Annual water balance	−0.23	0.09	0.13	0.14	0.12	−0.55	−0.63	−0.68	−0.68	−0.52	−0.52	−0.60	0.72	0.75	0.73	0.59	1.00	0.72	0.89	−0.14
Precip. coldest quarter	0.36	0.64	0.67	0.67	−0.44	−0.03	−0.16	−0.27	−0.28	−0.86	−0.86	−0.85	0.96	0.91	0.75	0.60	0.72	1.00	0.61	−0.52
Priestley-Taylor alpha	−0.32	0.03	0.07	0.08	0.21	−0.64	−0.72	−0.77	−0.78	−0.50	−0.51	−0.60	0.61	0.61	0.60	0.48	0.89	0.61	1.00	−0.09
Precip. seasonality	−0.30	−0.43	−0.45	−0.45	0.29	−0.08	−0.02	0.03	0.03	0.52	0.52	0.50	−0.48	−0.29	−0.00	0.05	−0.14	−0.52	−0.09	1.00

Supplementary Figure S3. Spearman correlation matrix for 20 climate variables extracted for the European pollen calibration samples.

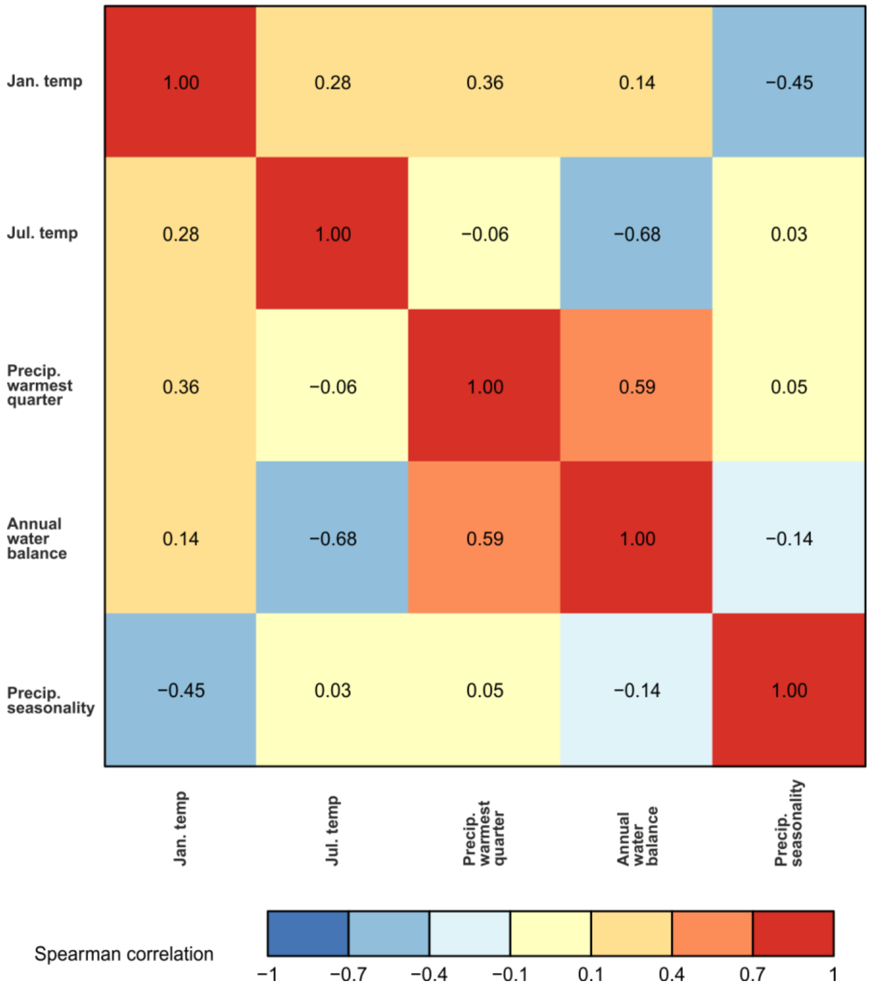

Supplementary Figure S4. Spearman correlation matrix for a subset of climate variables showing acceptable (absolute $\sigma < 0.7$) correlations in the European pollen calibration samples.

Supplementary Table S3. Coefficients of determination (R^2) for European pollen–climate models. For further details, see caption to Supplementary Table S1.

Rank	Variable	GAM	CTREE	RF	ETREES	EARTH	GAMBOOST	MBLACK	MGAM	ELM	NNET	Mean
1	**January temp.**	0.81	0.69	0.86	0.87	0.77	0.81	0.82	0.76	0.82	0.74	**0.79**
2	**July temp.**	0.71	0.57	0.81	0.83	0.70	0.72	0.76	0.69	0.68	0.68	**0.72**
3	**Annual water balance**	0.66	0.49	0.74	0.75	0.63	0.62	0.67	0.61	0.50	0.31	**0.60**
4	**Precip. warmest quarter**	0.55	0.28	0.62	0.62	0.46	0.50	0.58	0.45	0.35	0.18	**0.46**
5	**Precip. seasonality**	0.46	0.32	0.59	0.60	0.46	0.41	0.48	0.30	-3.28	0.30	**0.06**

Supplementary Table S4. European Nonmetric Multi-Dimensional Scaling results. For further details, see caption to Supplementary Table S2.

Rank	Variable	NMDS1	NMDS2	R^2
1	**January temp.**	-0.98	-0.21	0.56
2	**July temp.**	-0.53	0.85	0.52
3	**Annual water balance**	-0.04	-1.00	0.29
4	**Precip. warmest quarter**	-0.67	-0.74	0.15
5	**Precip. seasonality**	1.00	0.06	0.14

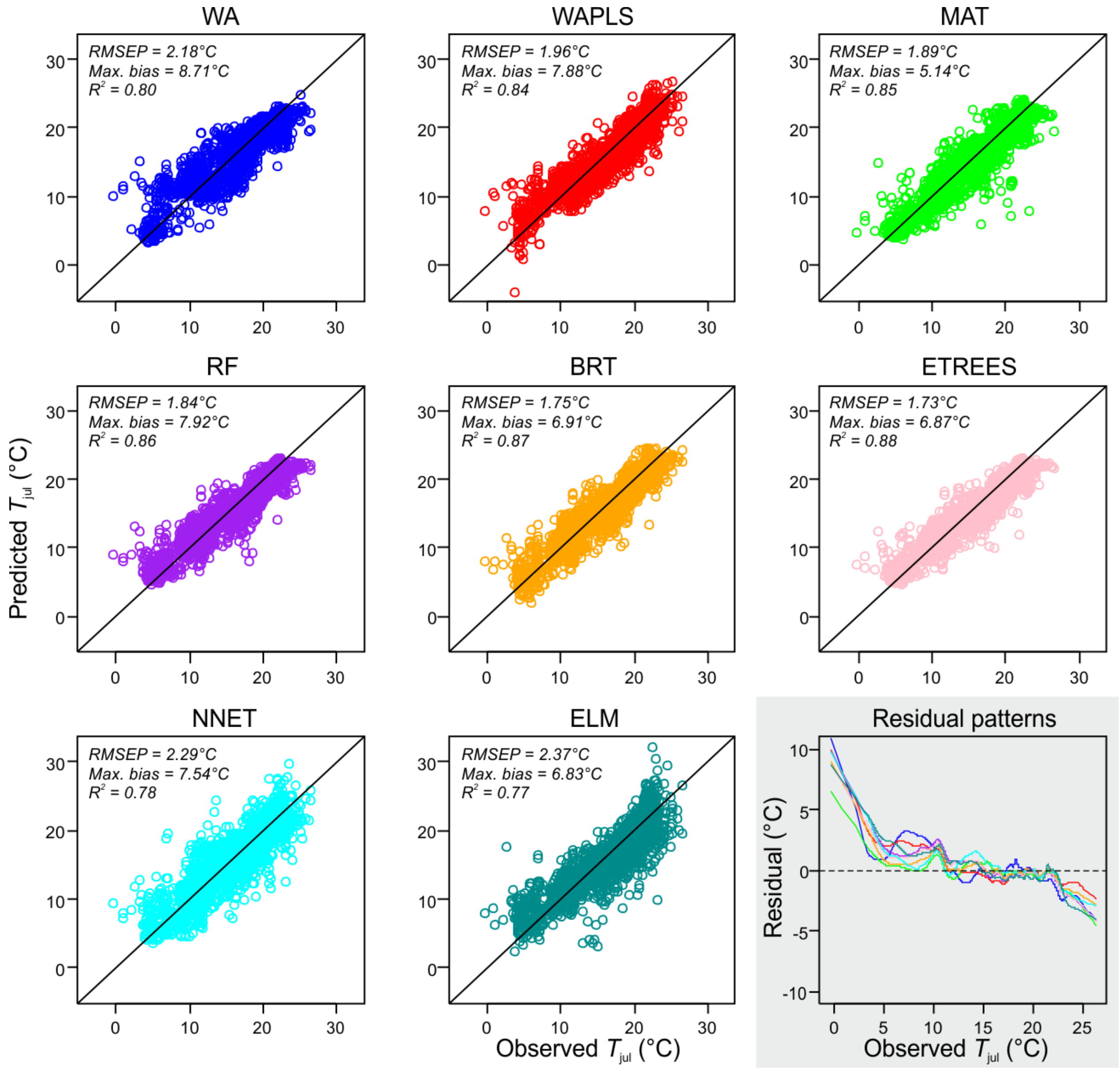

Supplementary Figure S5. Cross-validation results for North-American pollen–July mean temperature (T_{jul}) models, using an *h*-block cross-validation with a radius of 600 km. The first eight panels show the predicted vs. observed values of the eight calibration models considered. The model performance is summarized with the root-mean-square error of prediction (RMSEP), the maximum (max.) bias and the coefficient of determination (R^2). The final panel (*grey background*) shows the residual patterns of all eight models. Here, LOWESS smoothers (span=0.05) are fitted to the residuals of each model. The individual models are distinguished by the line colours which follow the predicted-vs.-observed value plots.

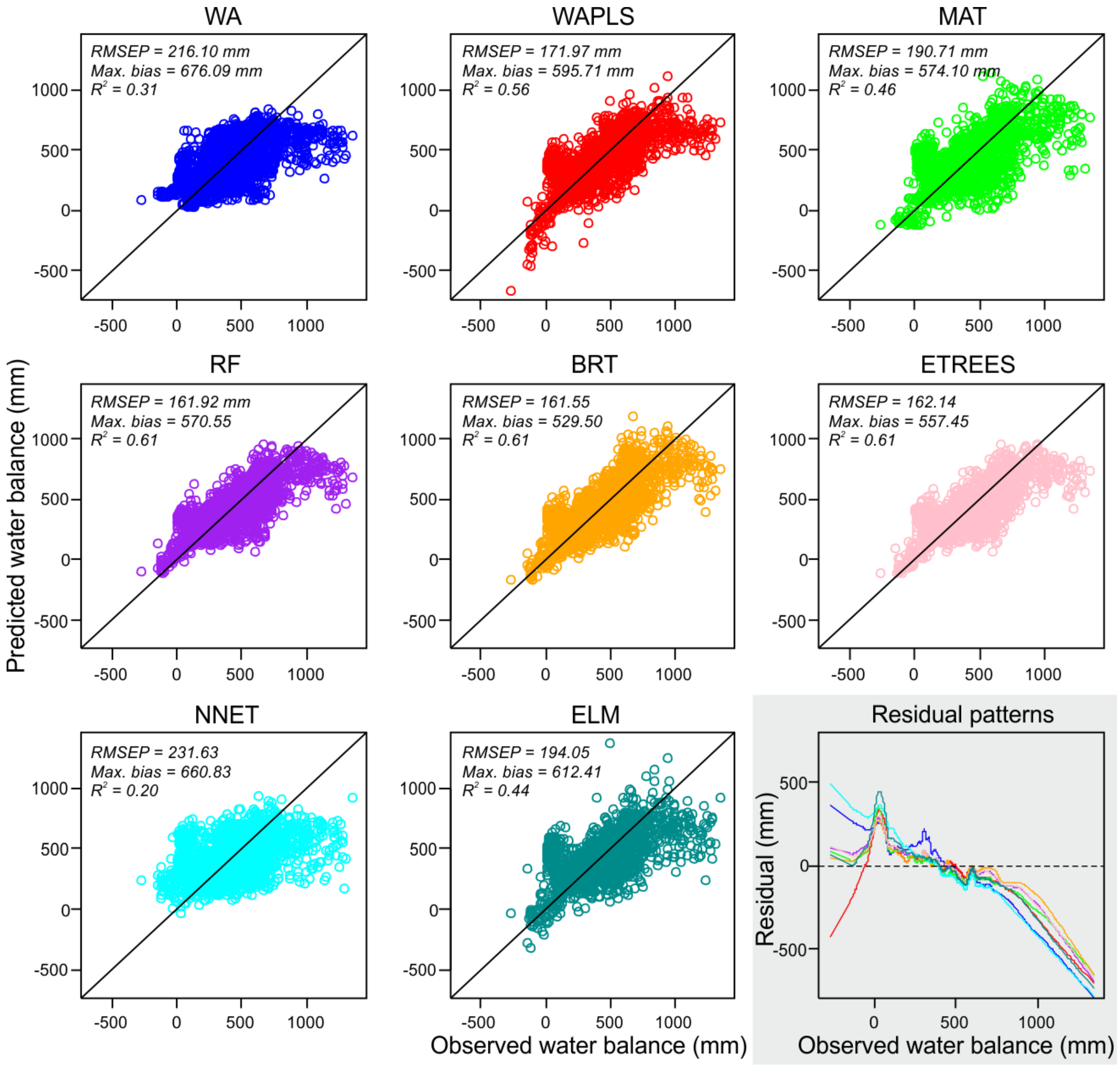

Supplementary Figure S6. Cross-validation results for North-American pollen–water balance models, using an *h*-block cross-validation with a radius of 400 km. For further details, see caption to Supplementary Figure S5.

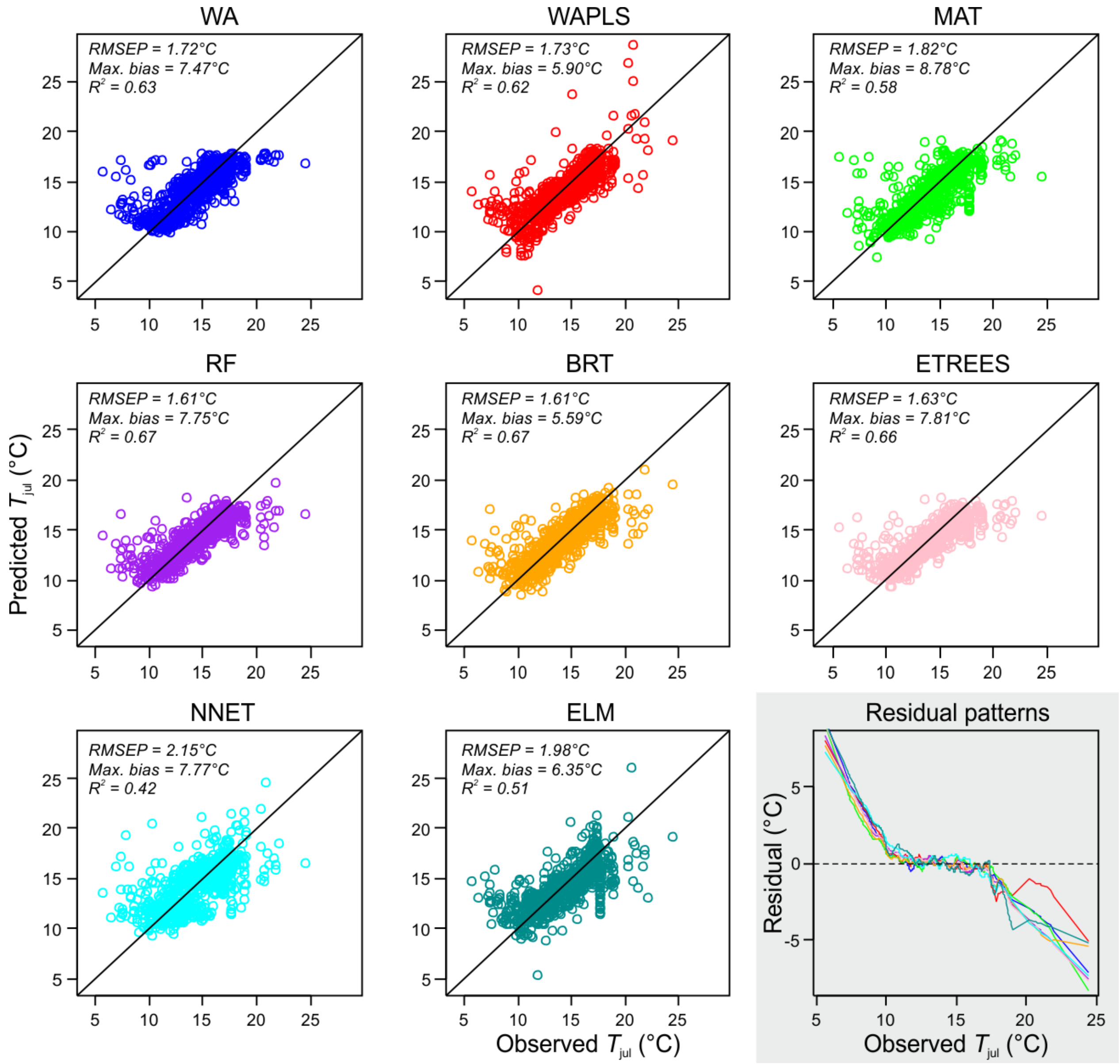

Supplementary Figure S7. Cross-validation results for European pollen–July mean temperature (T_{jul}) models, using an *h*-block cross-validation with a radius of 200 km. For further details, see caption to Supplementary Figure S5.

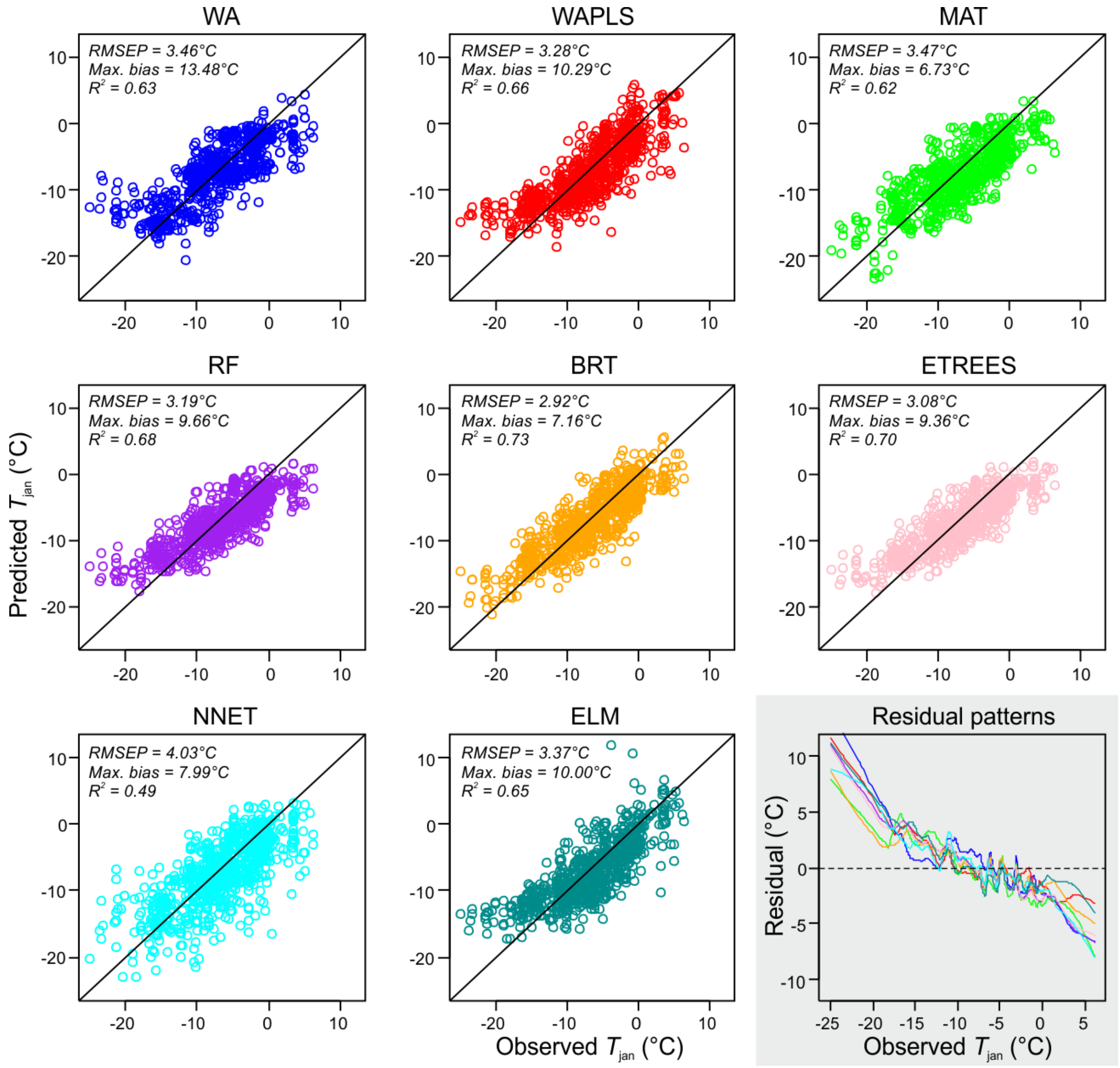

Supplementary Figure S8. Cross-validation results for European pollen–January mean temperature (T_{jan}) models, using an *h*-block cross-validation with a radius of 300 km. For further details, see caption to Supplementary Figure S5.

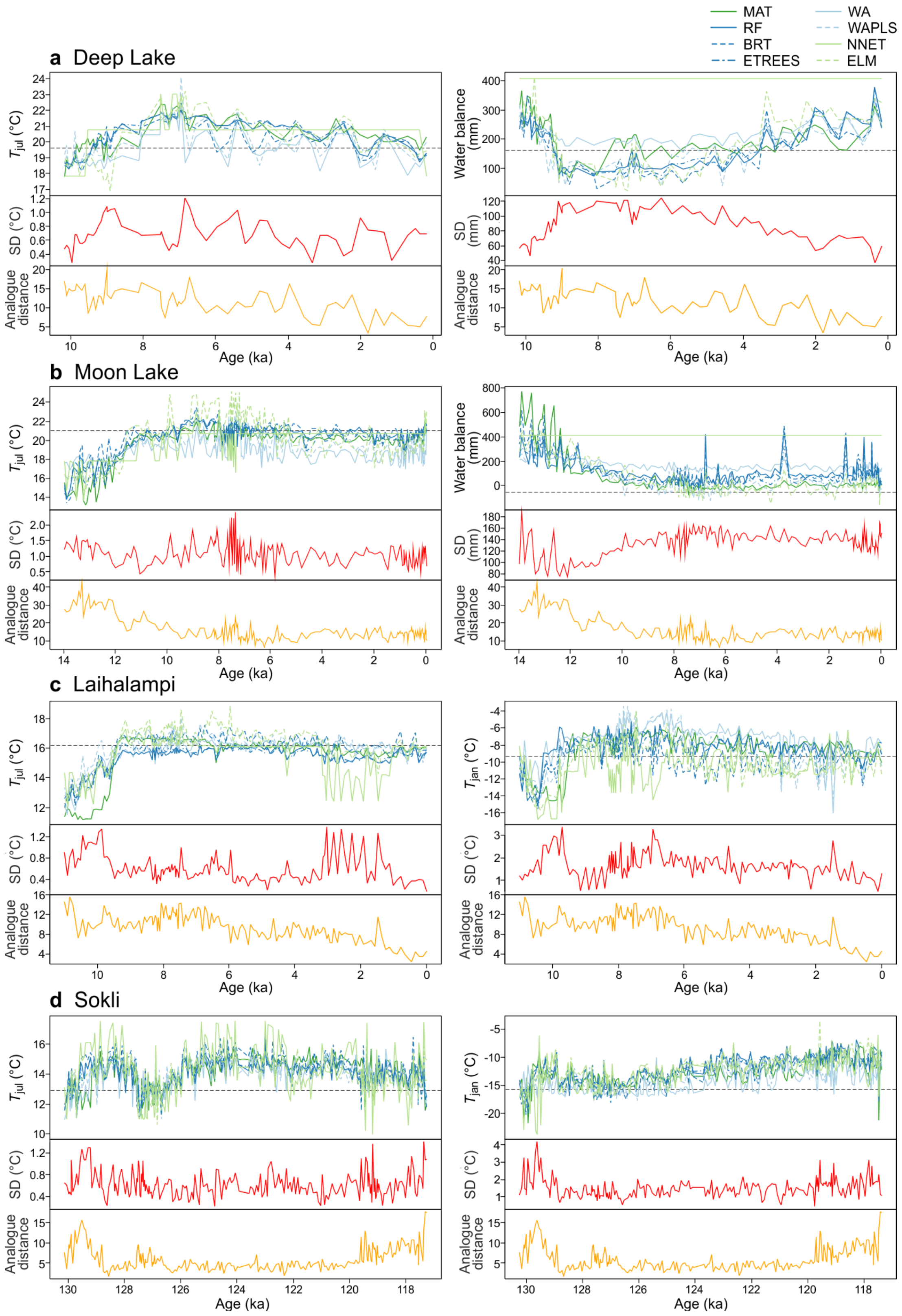

Supplementary Figure S9. Reliability metrics for the palaeoclimate reconstructions. Reconstructions (*blue* and *green* curves in the uppermost panels) are shown for primary and secondary climate variables and prepared with eight calibration methods from each fossil dataset. For each reconstruction, we show the standard deviation of the eight-method ensemble (*red*) and the compositional distances (squared chord distance) between the fossil pollen samples and the closest modern pollen analogues found in the pollen–climate calibration dataset (*orange*).
